# Supplementary material for: The anti-parasitic agent suramin and several of its analogues are inhibitors of the DNA binding protein Mcm10
Source: Open Biol. 2019 Aug 14;9(8):190117. doi: 10.1098/rsob.190117 (PMC6731595; doi:10.1098/rsob.190117)
Supplement: Supplementary data including 1 Table and 9 Figures [file rsob190117supp1.docx]

The anti-parasitic agent suramin and several of its analogs are inhibitors of the DNA binding protein Mcm10

Carolyn N. Paulson,^†^ Kristen John,^†^ Ryan M. Baxley,^‡^ Fredy Kurniawan,^‡^ Kayo Orellana,^‡^ Rawle Francis,^†^ Alexandra Sobeck,^‡^ Brandt F. Eichman,^+^ Walter J. Chazin,^§^ Hideki Aihara,^‡^ Gunda I. Georg,^†^ Jon E. Hawkinson,^†*^ and Anja-Katrin Bielinsky^‡*^

† Department of Medicinal Chemistry and Institute for Therapeutics Discovery & Development, College of Pharmacy, University of Minnesota, Minneapolis, MN 55414

‡ Department of Biochemistry, Molecular Biology and Biophysics, College of Biological Sciences, University of Minnesota, Minneapolis, MN 55455

+ Departments of Biological Sciences and Biochemistry, Center for Structural Biology, Vanderbilt University, Nashville, TN 37232

§ Departments of Biochemistry and Chemistry, Center for Structural Biology, Vanderbilt University, Nashville, TN 37240

| **Supplementary Material Table of Contents** | **Page** |
| --- | --- |
| **Materials and Methods** |  |
| Fluorescence polarization assay controls, hit confirmation, proteins, and data analysis | 1 |
| Screening collections, compounds, and reagents | 2 |
| Protein production and purification | 2 |
| Compound analysis by LCMS | 3 |
| SPR competition and control experiments | 3 |
| **Supplementary Figure S1.** Saturation binding curves for FP probe at Mcm10 constructs and RPA | 4 |
| **Supplementary Table S1.** Affinity values for FP probe at Mcm10 constructs and RPA | 4 |
| **Supplementary Figure S2**. Mcm10 FP HTS assay performance | 4 |
| **Supplementary Figure S3**. Competitive displacement curves of ssDNA by suramin analogs | 5 |
| **Supplementary Figure S4.** SPR sensorgram for suramin binding to a lower surface density of hMcm10-ID | 6 |
| **Supplementary Figure S5**. Mcm10 species differences for *iso*-PPADS and PPADS | 6 |
| **Supplementary Figure S6.** Representative SPR sensorgrams for suramin analogs at hMcm10-ID | 7 |
| **Supplementary Figure S7.** Correlation of SPR kinetic K_D_ values with the FP K_i_ values | 7 |
| **Supplementary Figure S8.** Comparison of binding models to NF546 SPR data | 8 |
| **Supplementary Figure S9.** Cytotoxicity of suramin analogs for hTERT-RPE1 and HCT116 cells | 8 |
| **References** | 9 |

**Materials and Methods**

Fluorescence polarization assay controls, hit confirmation, proteins, and data analysis

The LOPAC pilot screen identified the sulfhydryl reagent 4-chloromercuribenzoic acid as an inhibitor in the fluorescence polarization (FP) assay. As there was no known Mcm10 inhibitor, 4-chloromercuribenzoic acid (30 μM) was used as a positive control on initial screening plates. Following the discovery of suramin as a Mcm10 inhibitor, suramin (7 μM) was used as a positive control on subsequent screening plates. To confirm activity, screening hits were tested in dose response experiments (8 concentrations in duplicate) using xMcm10-ID. Similar dose response experiments were conducted with suramin and its analogs to confirm activity for hMcm10-ID (1 μM) and xMcm10-FL (2 μM) and to determine selectivity relative to RPA70AB (0.4 μM). Saturation experiments for each protein were conducted using increasing concentrations of protein with a fixed probe concentration (12.5 nM). FP was measured in mP units, and ∆mP values for each well were calculated as the difference between the measured mP and the average of the “probe only” controls. All raw mP values were corrected for background measurements, using the average of the buffer controls. ∆mP values from compound wells were then expressed as a percent of the average ∆mP values of control wells that did not contain any compaound. The parallel and perpendicular mP values were also used to calculate total fluorescence intensity (TFI) determined by the sum of parallel mP + (2 x perpendicular mP). TFI was used to detect fluorescence interference by compounds [1]. For the HTS, all compounds showing > 25% inhibition of the ∆mP signal and < 3x the standard deviation of the TFI were defined as hits. IC_50_ values were calculated using the four parameter logistic equation in Prism 6.0 (GraphPad). K*_i_* values were obtained using an online K*_i_* calculator according to the equations of Nikolovska-Coleska [2] (<http://sw16.im.med.umich.edu/soft-ware/calc_ki/>). K_D_ values were calculated using the one site or two site binding equations in Prism 6.0 (GraphPad).

Screening collections, compounds, and reagents

Suramin sodium salt was obtained from Sigma. NF023, NF110, NF157, NF279, NF340, NF449, NF546, pyridoxalphosphate-6-azophenyl-2’, 5’-disulfonic acid tetrasodium salt (iso-PPADS), and pyridoxalphosphate-6-azophenyl-2’, 4’-disulfonic acid tetrasodium salt (PPADS) were all purchased from TOCRIS. 5’-6FAM 10-mer DNA (5’-6-FAM-ATG GTA GGC A-3’) was obtained from Integrated DNA Technologies. Approximately ~154,000 compounds were screened, including the Library of Pharmacologically Active Compounds (LOPAC) (1280), NIH Clinical Collection 1 (446), Prestwick Chemical Library (1120), Tocris Tocriscreen (1120), Microsource Spectrum Collection (2000), Johns Hopkins FDA Collection (1514), NIH Clinical Collection 2 (281), Tocris Tocriscreen Kinase Inhibitor Toolbox (80), GSK (GlaxoSmithKline) Published Kinase Inhibitor Set (PKIS) Set 1 (367) and Set 2 (473), SelleckChem kinase inhibitor library (277), ChemDiv Tyrosine Kinase Targeted Library (8504), AnalytiCon NATx Natural Product-Based Library (5000), TimTec Natural Derivatives Library (NDL 3000) (3040), TimTec Privileged Structures Library (5470), National Cancer Institute (NCI) Library (2396), ChemDiv Peptidomimetic Library (6656), University of Minnesota (UMN) Legacy Collection (794), NCI Experimental Therapeutics (NeXT) Diversity Library (12078), ChemBridge DiverSet-EXP Library (50000), ChemBridge Diversity Library (46080), ChemDiv Polymerase Collection (1012), and the Life Chemicals Polymerase Collection (3976). These compounds were stored at ~10 mM in DMSO in 384-well plates.

Protein production and purification

xMcm10-ID (amino acid residues 229 to 427) and hMcm10-ID (residues 237 to 436) were expressed in *E. coli* strain BL21(DE3). The fragments were cloned into a modified pET28a vector with the human rhinovirus (HRV) 3C protease cleavage site following the N-terminal 6xHis-tag. Transformed bacteria were grown at 37 °C in LB medium to an OD_600_ of ~0.5, upon which isopropyl-β-D-1-thiogalactopyranoside (IPTG) and ZnCl_2_ were added to final concentrations of 0.5 mM and 50 μM, respectively. Protein expression was allowed to continue overnight at 18 °C. *E. coli* cells were pelleted at 4,500 x g for 20 min, resuspended in 50 mM Tris-HCl pH 7.4, 0.5 M NaCl, 5 mM imidazole, 10 mM β-mercaptoethanol, and lysed by the addition of 0.5 mg/mL (final) hen egg white lysozyme and sonication. Samples were then centrifuged at 64,000 x g for 1 hr. The cleared lysate was passed through a Ni-NTA column, which was subsequently washed with the same buffer containing 20 mM imidazole, and the bound protein was eluted with a linear imidazole concentration gradient up to 300 mM. The eluted protein was pooled, treated overnight with HRV 3C protease at 4 °C to remove the His-tag, and further purified by gel-filtration over a HiLoad 26/60 Superdex 200 column operating with the running buffer 20 mM Tris-HCl pH 7.4, 0.5 M NaCl, 10 mM β-mercaptoethanol. The full-length xMcm10 was expressed with MBP fused onto the N-terminus and 6x-His tag attached to the C-terminus of the protein [3]. Protein expression and purification were performed as described above, except that 100 μg/mL (final) RNaseA was added during the lysis step, the lysis buffer contained 1.0 M NaCl, and a Superdex 200 (10/300) column was used for gel-filtration. The MBP-tag was kept uncleaved as it helped with protein solubility and did not interfere with DNA-binding. The purified Mcm10 proteins were concentrated by ultrafiltration, flash frozen in small aliquots by liquid nitrogen, and stored at -80 °C. The protein concentrations were determined based on UV absorption at 280 nm and theoretical extinction coefficient calculated from the amino acid sequences.

Vector construction and production of recombinant human RPA70AB (181-422) in a pSV281 vector that incorporates an N-terminal 6xHis-tag containing a TEV cleavage site has been reported previously [4]. To summarize briefly, RPA70AB was expressed in the *Escherichia coli* host BL21(DE3) cells using Terrific Broth medium containing kanamycin at 37 °C. After cell lysis, the soluble fraction was run over a Ni affinity column, and then the 6xHis-tag was cleaved using TEV protease of RPA70AB. Further purification was achieved using heparin chromatography followed by Superdex-75 gel filtration. The final yield was 15-20 mg/liter of culture. The purity of the sample was confirmed using SDS-PAGE and electrospray mass spectrometry.

Compound purity analysis by LCMS

All purchased compounds were analyzed for purity by analytical LCMS. Compounds were dissolved in MilliQ water to make aqueous stock solutions ranging from 8-17 mM. The stock solutions were then diluted 500-fold using MilliQ water and filtered (0.2 μm, PTFE) prior to purity analysis using an Acquity UPLC (Waters Corporation) equipped with an Acquity BEH UPLC C18, 1.7 μm (2.1 x 50 mm) column for separation. Compound absorbance was detected at 214 nm using a photo diode array detector. Mass data was acquired using a Micromass ZQ mass spectrometer. LC was completed using a gradient method with each of the following mobile phase systems: Mobile Phase A2: 95% water, 5% acetonitrile, 10 mM ammonium acetate; Mobile Phase B2: 95% acetonitrile, 5% water, 10 mM ammonium acetate; Mobile Phase A1: 95% water, 5% methanol, 10 mM ammonium acetate; Mobile Phase B1: 95% methanol, 5% water, 0.1% 10 mM ammonium acetate. All compounds except iso-PPADS and PPADS were found to have > 95 % purity. PPADS had >91 % purity in mobile phase A1/B1 while iso-PPADS had > 90 % purity in mobile phase A2/B2.

SPR competition and control experiments

For ABA competition SPR experiments, the CM5 chip surface was prepared as described in Materials and Methods. Solution A was prepared with 50 μM NF023 in running buffer while solution B was prepared with the competitor near its K_D_ value (NF023 = 5 μM, suramin = 0.5 μM, and iso-PPADS = 30 μM) ± 50 μM NF023 in running buffer. Data were collected at a rate of 40 Hz and analyzed with the Biacore S200 Evaluation Software. For these experiments, hMcm10-ID was immobilized to the chip surface and NF023 at a concentration 10-fold higher than its K_D_. A running buffer control was performed to establish the baseline of the experiment. When buffer and NF023 were injected as solution B, there was a small decrease in RU due to slight buffer mismatch effects (Figure 5a). Buffer injected alone as solution B resulted in no response. NF023, suramin, and iso-PPADS were chosen as competitors to be tested at concentrations equal to their respective K_D_ values as determined by SPR.

Although the complex kinetics of NF546 is likely due to conformational selection and induced-fit conformational change, it is possible that the complex binding observed for NF546 by SPR is due to avidity effects that can often plague SPR experiments with higher density ligand surfaces. In an effort to explore whether this was the case, a lower density surface (3000 RU) of hMcm10-ID was prepared and suramin was chosen as a test compound. Similar *K*_D_ values were obtained for suramin between the high density and low density surfaces (Supplementary Figure S7). As such, it is unlikely that a lower density surface would greatly change the SPR results.


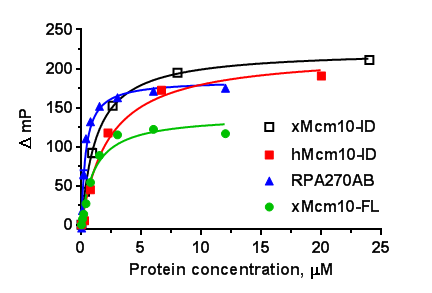


**Supplementary Figure S1**. Saturation binding curves for FP probe 5’-6FAM 10-mer ssDNA (12.5 nM) binding to human Mcm10 internal domain, *Xenopus* Mcm10 internal domain, full length *Xenopus* Mcm10, and Replication Protein A 70AB. Error bars represent ± SEM, where n ≥ 3 for all protein except xMcm10-FL. The xMcm10-FL curve represents the average of n=2.

**Supplementary Table S1**. Affinity of 5’-6-FAM 10-mer ssDNA for human Mcm10 internal domain (hMcm10-ID), *Xenopus* Mcm10 internal domain (xMcm10-ID), full length *Xenopus* Mcm10 (xMcm10-FL), and human Replication Protein 70AB (RPA70AB)

| protein | K_D_, μM^a^ |
| --- | --- |
| hMcm10-ID | 2.29 ± 0.02 |
| xMcm10-ID | 1.3 ± 0.1 |
| xMcm10-FL | 1.1 (0.8 - 1.3)^b^ |
| RPA70AB | 0.37 ± 0.03 |

^a^Mean ± SEM, where n = 3. ^b^Mean (95% confidence interval); n = 2 for xMcm10-FL protein due limited protein availability.


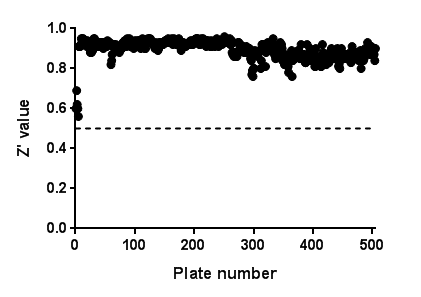


**Supplementary Figure S2.** FP HTS assay performance. Z’ values in the pilot screen using the 3’-6FAM probe averaged 0.61, which increased to an average of 0.90 for the main HTS using the 5’-6FAM probe (average Z’ value for the entire screen was 0.89). All plates had a Z’ value > 0.5 (dotted line).


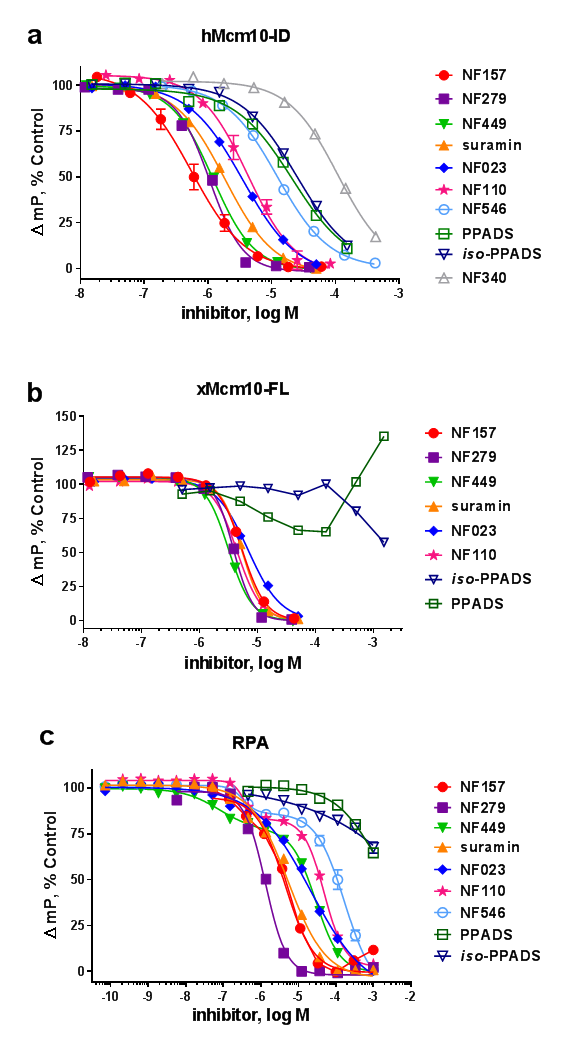


**Supplementary Figure S3**. Competitive displacement of ssDNA by suramin analogs from (a) human Mcm10 internal domain (hMcm10-ID), (b) full length *Xenopus* Mcm10 (xMcm10-FL), and (c) human Replication Protein A 70AB (RPA70AB) measured by FP. Note the inactivity of PPADS and *iso*-PPADS at *Xenopus* Mcm10 and the two component displacement curves for NF449, NF110, and NF546. Error bars for hMcm10 and RPA70AB represent ± SEM where n ≥ 3. xMcm10-FL displacement curves represent the average of two independent experiments. The tailing up of the *iso*-PPADS dose-response curve at concentrations of ~ 1 mM in panel B could be due to lack of solubility, a detergent-like effect of this amphipathic molecule, or fluorescence interference.





**Supplementary Figure S4.** SPR sensorgram of suramin binding to immobilized human Mcm10 ID (hMcm10-ID) with a lower protein density. A kinetic K_D_ = 0.65 μM obtained using 3000 RU of protein immobilized on the chip surface is comparable to the K_D_ = 0.57 μM obtained from the high density surface (6000 RU).


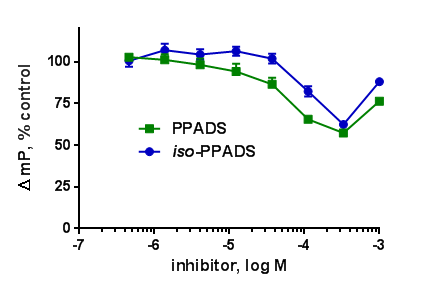


**Supplementary Figure S5**. *iso*-PPADS and PPADS weakly and incompletely inhibit the binding of the 5’-6FAM probe to *Xenopus* Mcm10 internal domain (xMcm10-ID), indicating a species difference between *Xenopus* and human proteins. Compare to the activity of these inhibitors with hMcm10-ID (Fig. 3a) and xMcm10-FL (Fig. 3b). Error bars represent ± SEM where n ≥ 3. The tailing up of the dose-response curves for both compounds at concentrations of 1 mM could be due to lack of solubility, a detergent-like effect of these amphipathic molecules, or fluorescence interference.

**

**

**Supplementary Figure S6**. Representative SPR sensorgrams of NF 449, NF110, NF 157, NF279 and PPADS binding to immobilized human Mcm10 internal domain (hMcm10-ID). n > 3. Note the predominantly rapid kinetics of NF 449, NF110, NF 157, and NF279 compared to the slow association and dissociation kinetics of PPADS.


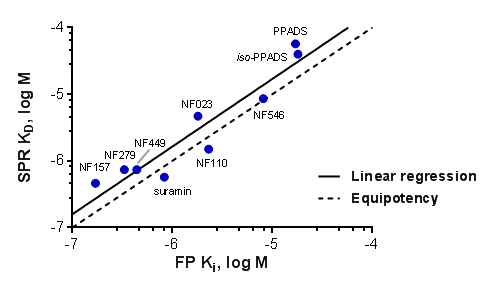


**Supplementary Figure S7.** SPR kinetic K_D_ values correlate well with the K*_i_* values obtained by fluorescence polarization. r^2^ = 0.89.


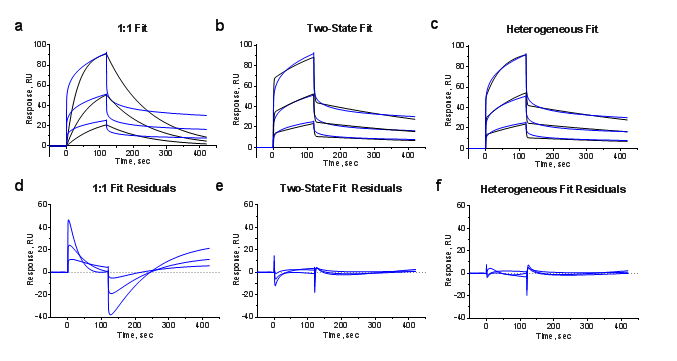


**Supplementary Figure S8.** NF546 binds to Mcm10 with complex kinetics. A comparison between the (a) 1:1 binding model (χ^2^ = 180 ± 47), (b) two-state binding model (χ^2^ = 4.2 ± 1.0), and (c) heterogeneous ligand binding model (χ^2^ = 3.2 ± 0.9) fits (black) to the SPR sensorgrams (blue) of NF546 and their respective residuals (difference between the experimental data and fitted curves) (d-f) show that the 1:1 binding model fits the experimental data poorly. χ^2^ values are means ± SEM of the goodness of fit of the model to the experimental data for the three concentrations of NF546 common to all three experiments (5.6,17.6, and 50 μM). The fits to the two-state conformational change and heterogeneous ligand (i.e., > two populations of protein immobilized on the sensor chip) models are significantly better than the 1:1 Langmuir model, but are not different from each other (p = 0.0059, ANOVA and Tukey’s multiple comparisons test).


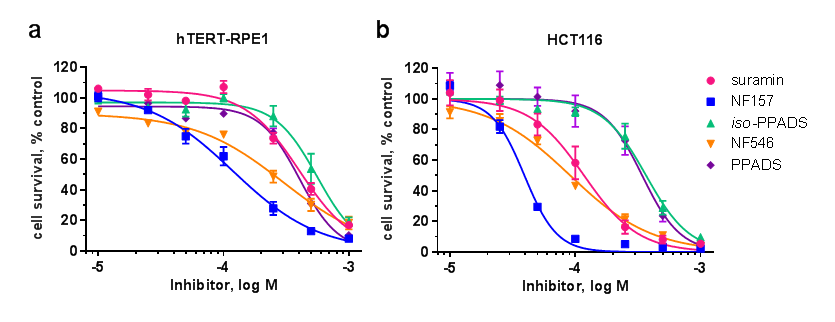


**Supplementary Figure S9.** Cytotoxicity of suramin analogs for (a) normal epithelial hTERT RPE-1 cells (b) colon cancer HCT116 cells determined by measuring intracellular ATP concentrations. Error bars represent ± SEM where n = 3.

**References**

1 Turconi, S., Shea, K., Ashman, S., Fantom, K., Earnshaw, D. L., Bingham, R. P., Haupts, U. M., Brown, M. J. B., Pope, A. J. 2001 Real Experiences of uHTS: A prototypic 1536-well fluorescence anisotropy-based uHTS screen and application of well-level quality control procedures. *J. Biomol. Screen.* **6**, 275-290. (10.1177/108705710100600502)

2 Nikolovska-Coleska, Z., Wang, R., Fang, X., Pan, H., Tomita, Y., Li, P., Roller, P. P., Krajewski, K., Saito, N. G., Stuckey, J. A.*, et al.* 2004 Development and optimization of a binding assay for the XIAP BIR3 domain using fluorescence polarization. *Anal. Biochem.* **332**, 261-273. (doi.org/10.1016/j.ab.2004.05.055)

3 Robertson, P. D., Warren, E. M., Zhang, H., Friedman, D. B., Lary, J. W., Cole, J. L., Tutter, A. V., Walter, J. C., Fanning, E., Eichman, B. F. 2008 Domain architecture and biochemical characterization of vertebrate Mcm10. *J. Biol. Chem.* **283**, 3338-3348. (10.1074/jbc.M706267200)

4 Arunkumar, A. I., Stauffer, M. E., Bochkareva, E., Bochkarev, A., Chazin, W. J. 2003 Independent and coordinated functions of replication protein A tandem high affinity single-stranded DNA binding domains. *J. Biol. Chem.* **278**, 41077-41082.
